# Urea–Formaldehyde Strengthened by Polyvinyl Alcohol: Impact on Mulch Film Properties and Cucumber Cultivation

**DOI:** 10.3390/polym17091277

**Published:** 2025-05-07

**Authors:** Tingting Shen, Yongjie Ma, Xueyan Zhang

**Affiliations:** College of Enology and Horticulture, Ningxia University, Helanshan Xilu No. 489, Yinchuan 750021, China; tingshen2018@163.com (T.S.);

**Keywords:** polyvinyl alcohol-modified urea–formaldehyde, biocomposite, sprayable polymeric film, cucumber cultivation

## Abstract

To address the problem of environmental pollution caused by the extensive use of low-density polyethylene (LDPE) mulch film, this study developed a novel sprayable mulch using natural fibers and biodegradable polymers. Urea–formaldehyde resin (UF), strengthened with polyvinyl alcohol (PVA), was used as a modifier to induce beneficial physicochemical structural changes in PVA-modified urea–formaldehyde (PUF) resins. Characterization of these resins was conducted using Fourier transformation infrared spectroscopy (FT-IR), thermogravimetric analysis (TGA), and scanning electron microscopy (SEM). Preparation of the biodegradable mulch was conducted using Xuan paper waste residue (XP) as an enhancer, with PUF as the auxiliary agent. The resulting film (PUF-XP) was examined for differences in thickness, morphological characterization, and rate of weight loss, and the effects of different covering films on cucumber growth, root development, soil temperature, and weed control were evaluated. Characterization reveals that when the PVA content was 4% (W4UF), the film had the lowest free formaldehyde content (0.26%) and highest elongation at break (5.70%). In addition, W4UF could easily undergo thermal degradation at 278.4 °C and possessed a close-knit, three-dimensional structural network. W4UF was then mixed with paper powder and water in various proportions to produce three mulch films (BioT1, BioT2, and BioT3) that demonstrated excellent water retention and heat preservation and inhibited weed growth by 68.8–96.8%. Compared to no mulching (NM), BioT1 increased both the specific root length and root density, as well as improved the plant height, stem diameter, and total biomass of the cucumbers by 43.5%, 34.1%, and 33.9%, respectively. Therefore, a mass ratio of paper powder, water, and W4UF of 1:30:2 produced a biodegradable mulch film that could be used as an alternative to LDPE, mitigating the environmental pollution rendered by synthetic plastic mulch films and offering the potential for a sustainable agricultural application.

## 1. Introduction

Plastic mulching films have been widely used in agricultural production, serving as an essential measure for increasing food production and security in response to global population increases [1,2]. These films benefit crops by creating favorable micro-climates that prevent weed growth and increase soil temperature and humidity, effectively increasing crop yield [3]. In recent years, low-density polyethylene (LDPE) has become a primary material used for soil mulching due to its excellent chemical stability and processability [4]. However, LDPE mulch has difficulty degrading naturally, and the use of significant quantities worldwide results in residual plastic pollution that poses a threat to global ecology [5]. Therefore, for agricultural advancement, it is essential to develop novel biodegradable mulches from renewable raw materials [6]. In recent years, there has been great interest in the use of biomass, lignin, and cellulose waste to produce high-value-added composites [7]. Some scholars have proposed using waste from agricultural production, such as packaging boxes, covering coatings, and degradable flower pots [8,9]. As a raw material, waste biomass must be treated and mixed with an adhesive in a particular ratio to prepare a composite material. The mulch film can then be formed by spraying the mixture over the soil, offering the same covering effect as traditional mulch film [1,10]. At present, based on biodegradable materials such as starch, cellulose, chitosan, alginate, and glucomannan [11] and combined with hydroxyethyl cellulose, polyvinyl alcohol, and lignocellulose waste, liquid biodegradable mixtures are prepared by chemical processes, and biodegradable coating films are formed in the field by hot film forming, casting, or spraying technology [12]. Biodegradable mulch films can be applied according to the crop cultivation cycle or growth needs, and the biofilms are degraded by microorganisms in the soil after harvest, effectively improving the soil quality and facilitating ecosystem restoration. Thus, they may be considered an attractive alternative to LDPE films [13].

Sprayable agricultural mulch suspensions were prepared from sodium alginate and microparticles of the seaweed Undaria pinnatifida (A) at concentrations of 0.5, 1, and 2 wt%. By forming a biodegradable mulch on the soil in situ, the material reduced water loss, increased soil temperature, promoted plant growth, and triggered the growth of beneficial soil microbes [14]. Composites prepared from horn powder, mixed with crude glycerol and polyvinyl alcohol at mass ratios of 0.75, 1.5, and 2.25 as the raw materials, were evaluated for the effect of glycerol dosage and horn powder particle size on the mechanical and adsorption properties, air permeability, and the water-soluble nitrogen and carbon content of the composite films. The liquid composite was then sprayed on the surface of potted plants to form a polymer coating, and the covering film was shown to retain moisture, promote crop growth, inhibit weeds, and stimulate microbial activity in the soil [15]. However, although these coatings demonstrated ease of application, they exhibited poor mechanical properties and low elongation at break after spraying and could not form a complete and consistent film, which limited their large-scale application. Therefore, it is critical to improve the mechanical properties of sprayable mulch films [16].

Due to the simple synthesis process, excellent thermal properties, low curing temperature, low cost, and excellent mechanical properties, urea–formaldehyde (UF) resin has been widely used as a wood adhesive [17]. Specifically, UF contains abundant amine, amide, hydroxyl, and carbonyl groups, which can be grafted with other polymers to form a stable three-dimensional structural network that improves the mechanical properties of materials [18]. UF has also been used as an adhesive for composite materials and as a slow-release fertilizer to improve soil fertility [19]. However, poor water resistance and high brittleness of the adhesive layer, as well as a high free formaldehyde content that can pose a threat to human health, severely limit UF’s practical application [20]. Resin modification or the development of new adhesives can both improve UF performance and reduce free formaldehyde content. Free formaldehyde in UF comes from the decomposition of methylene and other bonds, and when added as a modifier to the resin, it can reduce methylene and ether bonds [21]. Numerous studies have been conducted on UF modification to improve its durability. One method reduced the formaldehyde-to-urea (F/U) ratio, which reduced the degree of cross-linking and resulted in low mechanical strength and poor water resistance of the resin [22]. Another method used resin modifiers, such as phenols, tannin, lignin [23], melamine [24], and protein hydrolysate [25]; however, most UF modifiers are relatively expensive and can be toxic. Polyvinyl alcohol (PVA) is a water-soluble synthetic polymer rich in hydroxyl groups and has good tensile strength and flexibility. Due to the high activity of the side hydroxyl functional groups, PVA readily reacts with aldehydes, isocyanates, and anhydrides [26]. Moreover, PVA has shown good adhesive qualities, which can improve the viscosity and bonding strength of resins, allowing it to be used to prepare UF with low formaldehyde content and suitable viscosity [27]. A large number of these hydroxyl groups will also self-associate with formaldehyde during the synthesis process through hydrogen–bond interactions, enabling the modified UF to capture residual formaldehyde and form a PVA-modified UF resin (PUF) interpenetrating network [28].

Biodegradable mulch requires good durability and suitable mechanical strength. The addition of fillers such as fibers (natural plant fibers, glass fibers, and mineral fibers) to the composite material can effectively improve the performance of the material [29,30]. The primary raw materials of xuan paper (XP) include Shatian straw and Pteroceltis tatarinowii bark, where a combination of the two is conducive to the mutual weaving of fibers. XP residues are the solid waste produced during production, and currently, about 1000 tons are produced every year in China, and over 2000 tons of XP residues are discharged, occupying considerable land and resulting in a wasted resource [31]. Thus, it is desired to adapt the research that used ordinary paper waste to prepare composite membranes and employ XP as reinforcing fillers for mulch films [32].

The objectives of this work were to improve the plasticity of UF using PVA and explore the effect of PVA as a modifier on the free formaldehyde content, mechanical properties, and durability of UF composites. The biodegradable mulch film (PUF-XP) was prepared using PUF as the auxiliary agent and XP as a reinforcing filler, and the film was formed by a spraying process that was applied during cucumber cultivation. The specific aims were to (1) quantify the PVA modification of UF structural changes toward the durability of the polymer, (2) explore the properties of the PVA, UF, and PUF-XP blend systems, as well as the mulch film, and (3) conduct a comprehensive evaluation of the impact of PUF-XP on cucumber growth, plant biomass, and the root system.

## 2. Materials and Methods

### 2.1. Materials

Formaldehyde (37 wt% aqueous solution), PVA, sodium hydroxide, ammonium chloride, acetic acid, and sodium carbonate were purchased from the Xilong Science Company (Shantou, China). Urea was purchased from Guangdong Guanghua Technology Co., Ltd. (Shantou, China). Sodium sulfite, boric acid, iodine, and potassium iodide were purchased from the Tianjin Comeo Chemical Reagent Company (Tianjin, China). Raw XP residues were purchased from Beijing Ziguang Yingli Chemical Technology Co., Ltd. (Beijing, China), where the main components were a mixture of cellulose, hemicellulose, and lignin at a pH of 5.5–8.5, total nutrient content ≥ 6%, organic matter content ≥ 50%, and humic acid content ≥ 30%. The raw residue was air-dried and crushed into a 300-mesh powder. The cucumber seedlings consisted of Bome 626 varieties and were purchased from the Tianjin Derite Seed Company (Tianjin, China).

### 2.2. Preparation of PUF

The modified urea–formaldehyde resin was prepared by the alkali–acid–alkali method (molar ratio F:U = 1.4:1), where 37% formaldehyde solution was added to a three-necked flask, and the solution was heated to 40 °C under stirring. The pH of the resin was adjusted to 7.5–8.0, and the first batch of urea (F:U_1_ = 2:1) was added and allowed to react for 10 min. Afterward, the solution was heated to 60–65 °C, PVA was added, and heating was continued to 90 °C and held for 60 min. Then, the pH was adjusted to 4.8–5.1, and the reaction was allowed to continue to completion, at which time the pH was adjusted to 7.5–8.0. The second batch of urea [F:(U_1_ + U_2_) = 1.5:1] was then added and allowed to react at 80–85 °C for 30 min. The third batch of urea [F:(U_1_ + U_2_ + U_3_) = 1.3:1] was then added and allowed to react at 70–80 °C for 30 min. Finally, the pH was adjusted to 7.5–8.0, and the solution was allowed to cool to 40–50 °C. The PVA amounts were designed for 0%, 2%, 4%, 6%, and 8% of the total mass of urea and expressed as UF, W2UF, W4UF, W6UF, and W8UF, respectively.

### 2.3. Determination of Basic Performance of PUF

The pH, viscosity, solid content, curing time, free formaldehyde content, and water absorption were determined according to the GB/T14074-2006 standard-Testing methods for wood adhesives and their resins (National Forestry and Grassland Administration, China, 2006) [33], where the viscosity was measured using an NDJ-5S type viscometer. The mechanical properties of PUF were evaluated using ordinary filter paper as the carrier. The filter paper was cut into 18 × 1.5 cm strips and soaked in PUF under various treatments for 5 min, allowed to air dry indoors in a ventilated place, and then oven-dried at 90 °C for 40 min. The tensile strength and elongation at the breaking point of the impregnated filter paper were measured using an HY-0580 electronic (Shanghai Hengyi Precision Instrument Co., Ltd., Shanghai, China) universal mechanical testing machine [34].

### 2.4. Sample Characterization

The PUFs were freeze-dried for preservation using an Alpha1-4/2-4LD Plus vacuum freeze dryer and then ground into a powder. Molecular-level changes in UF during modification were determined by Fourier transform infrared spectroscopy (FT-IR) (Nicolet iS50-Thermo Scientific, Waltham, MA, USA) using 32 scans over a wavenumber range of 4000–500 cm^−1^ at a resolution of 4 cm^−1^. A thermogravimetric analyzer (EXSTAR series TG7200, SII NanoTechnology Inc., Tokyo, Japan) was used to evaluate the thermal properties of the UF structure, where the samples were heated from 30 °C to 600 °C at a heating rate of 10 °C/min under an N_2_ atmosphere. The various samples were gold-sputtered for 10 min and inspected using an Evols10 scanning electron microscope (Carl Zeiss AG, Oberkochen, Germany).

### 2.5. Preparation of Biodegradable Film

Excess W4UF was prepared and mixed with paper powder and water at powder/water/PUF ratios of 1:30:2, 1:30:4, and 1:30:6 to generate biodegradable composite materials BioT1, BioT2, and BioT3, respectively. First, the mixed hybrid solution was added to a multi-functional reactor, where it was heated to 60 °C and held for 30 min under constant stirring. The solution was then poured into a culture dish with sand at the bottom and placed in a drying oven at 30 °C for 48 h, resulting in a solidified biodegradable film.

### 2.6. Biodegradable Mulch Performance

Potting soil was passed through a 1 mm sieve and dried to constant weight at 105 °C. Eighty grams of dried soil were placed into a 9 cm diameter Petri dish, sprayed with 30 g of water, and covered with a 9 cm diameter piece of biodegradable mulch film. The weight was measured and recorded as m_0_. The dishes were then cultivated in a climate-controlled chamber for three days at 60% relative humidity and 28 °C, with 16 h of light per day. The dish weight was measured every two hours and recorded as mi. The percentage of mass loss was used to represent the water retention of the mulch film and was calculated as:

(1) $$m=(m_{0}-m_{i})/30\times 100\%$$

where m denotes the mass loss rate, m_0_ is the original weight, and mi is the weight after i hours (i = 2, 4, 6…).

All biodegradable mulching films were also placed in a textile bag and buried 15 cm below the ground surface for 120 days, where the soil moisture content was maintained at 60–90%, and the temperature was between 5 and 15 °C. The initial weights of the films were recorded as M_0_, and the films’ weights were recorded as M_1_ every 30 days after burial. The degradation ratio was calculated according to the formula:

(2) $$W=(M_{0}-M_{1})/M_{0}\times 100\%$$

where *W* denotes the degradation rate, M_0_ is the initial weight, and M_1_ indicates the weight after burial in the ground for a certain period.

### 2.7. Spraying of the Biodegradable Materials

Two days after transplanting the cucumber plants, BioT1, BioT2, and BioT3 were sprayed on the surface of the soil, where the quantity of sprayed solution determined the lifespan of the coating. Spraying was carried out at a coverage rate of 4.75 kg/m^2^, with a spray gun caliber of 2.25 mm and an air pressure of 1.2 MPa. The dry coating obtained was continuous and regular, with a thickness of 1.32–1.76 mm. No mulch and LDPE mulch films were used as controls. Soil temperature monitors (QM-ZC-16, Shangqiu Sensor Technology Co., Ltd., Shangqiu, China) were installed at a depth of 15 cm in the middle rows of various plots, and the temperatures that were recorded on a memory card every 30 min during the growing season were also used to calculate mean daily soil temperatures.

### 2.8. Bioassay Under a Greenhouse

The experiment was carried out from 28 November 2019, to 16 January 2020, in a north–south-oriented solar greenhouse on an experimental farm at the University of Ningxia, Yinchuan, China (38.26° N, 105.53° E). At an altitude of 1112 m, Yinchuan has a mid-temperate arid climate with annual solar radiation of 5723–6087 MJ/m^2^, temperatures of 6.3–13.1 °C, and a yearly rainfall of 133–589 mm. The experiment was carried out using a completely randomized block design with three replicates, where the plot size was 0.7 × 0.4 m (0.28 m^2^) with plant and row spacings of 20 cm, meaning each plot had six cucumber plants. Each plot also had the same amounts of applied fertilizer and irrigation. Base fertilizer included 45 g/m^2^ of diammonium and 75 g/m^2^ of compound fertilizer (N:P:K = 20:20:20), and the water and fertilizer management was unified for all treatments.

After transplantation, plant height and stem diameter were measured every week for each treatment. After the last measurement, the roots and fresh shoots of the six plants from each treatment group were weighed and dried at 80 °C to a constant weight. An EPSON V700 scanner (Seiko Epson Corporation, Suwa City, Japan)was used to assess the root morphology, with the root length, surface area, average diameter, and volume determined using the analysis software Win RHIZO (Regent instruments lnc., Québec, QC, Canada). The root soil volume was 4600 cm^3^, while the root length density, surface area density, and volume density were assayed by the ratio of root length, surface area, volume, and soil volume, respectively. The relative growth rates of the plant height (RGH-PH) and stem diameter (RGH-SV) were calculated as:

(3) $$\text{RGH-PH}=(\ln(h_{2})-\ln(h_{1}))/(t_{2}-t_{1})$$

(4) $$\text{RGH-SV}=\left(\ln({{d_{2}d}_{2}h}_{2})-\ln(d_{1}{d_{1}h}_{1})\right)/(t_{2}-t_{1})$$

where *h*_2_ and *h*_1_ represent the plant heights at *t*_2_ and t_1_, respectively, and *d*_2_ and *d*_1_ denote the stem thickness at *t*_2_ and *t*_1_, respectively.

### 2.9. Statistical Analysis

The data were statistically analyzed using Excel 2016 and SPSS 24.0, while the mean values were analyzed by the post hoc tests used for multiple comparison one-way ANOVA at a significance level of *p* < 0.05. Charts were generated using Origin 2018 (OriginLab lnc., Northampton City, MA, USA) and PowerPoint 2016 software (Microsoft lnc., Redmond City, WA, USA).

## 3. Results and Discussion

### 3.1. Basic Characteristics of PUF

After adding PVA, the pH and water absorption rate of the modified UF were significantly improved (Table 1), complying with GB/T 4897-2015-Particle board (China Academy of Forestry Wood Industry Research Institute, China, 2015). The viscosity increased with increasing PVA content, going from 23.67 mPa·s to 67.93 mPa·s at a PVA content of 4%. This was likely due to the adhesive qualities of the PVA caused by hydrogen bond interaction between -OH and water molecules in the PVA and N-H and OH in the UF. PVA participates in UF reactions by molding the cross-linking of the complex UF structural network, which increases the viscosity [35]. However, when the PVA reached 8%, excessive precipitation of crystals led to a decrease in viscosity. The addition of PVA also reduced the solid content of UF due to the reaction between PVA and UF, which generates formaldehyde and water, creating mass loss when the formaldehyde evaporates. Further, free formaldehyde content decreased with increasing PVA when the addition was below 4%. This occurred because PVA has more reaction sites than urea; however, excess PVA increased the viscosity of UF and formed a gel, leading to an increase in free formaldehyde [33]. Increased PVA content also lengthened the curing time of the PUF, possibly reflecting the reactivity of UF. PUF activity may have been reduced due to the steric hindrance of the PVA macromolecular chain and the decrease of active groups in the system [36].

### 3.2. Micro-Structural Characterization of PUF

**Figure 1:**
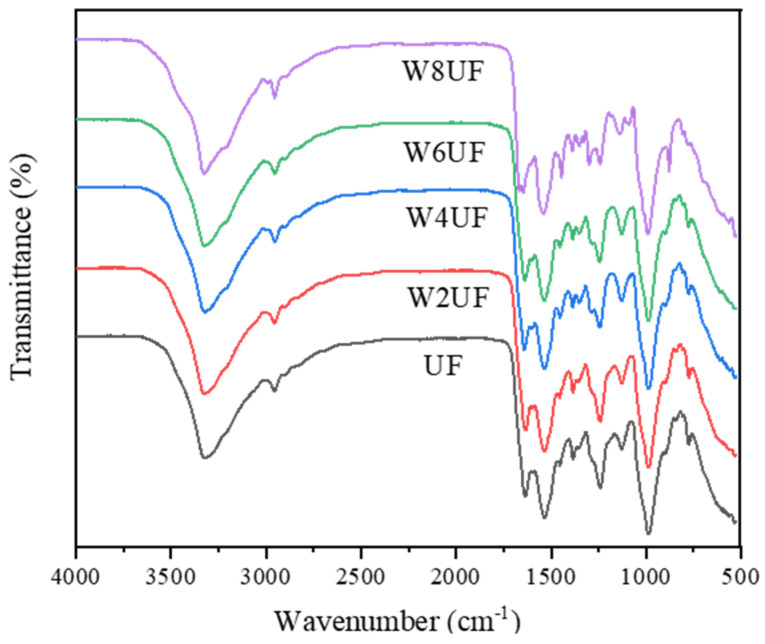
Fourier transform infrared spectroscopy (FT-IR) spectra of the modified urea–formaldehyde resin, where UF, W2UF, W4UF, W6UF, and W8UF indicate treatments representing PVA additions in urea of 0%, 2%, 4%, 6%, and 8%, respectively.

The FTIR spectra of PUF for the different treatment groups were highly similar (Figure 1), and the strong absorption peak at 3320 cm^−1^ corresponded to the O-H and N-H functional groups [37]. PVA had a strong absorption peak at O-H stretching vibrations, which suggests that increased PVA improved the bonding strength of the urea–formaldehyde resin [38]. The C-H symmetric stretching vibration absorption peak at 2953 cm^−1^ for W8UF was enhanced compared to the other treatments. The increase in the C-H group indicated increased free formaldehyde content in UF, which was consistent with the measurement results in Table 1. The strong absorption peak at 1631 cm^−1^ consisted of the stretching vibration of C=O in amide I, while the peak at 1534 cm^−1^ corresponded to the -NH bending vibration peak in amide II [38]. The absorption peaks at 1449 cm^−1^ and 1384 cm^−1^ were the bending vibration of the -CH_2_- and -NH_2_- functional groups, respectively; the stretching vibration peak of the C-N functional group was located at 1246 cm^−1^; and the absorption peak at 1135 cm^−1^ consisted of the asymmetric stretching vibrations of the -N-CH_2_-N group. The peak at 997 cm^−1^ was broad and robust, where the C-O stretching vibration peak correlated to the O-H and ether bonds in the hydroxymethyl group [39]. A weak absorption peak at 899 cm^−1^ was attributed to the bending vibrations of C-H for unsaturated hydrocarbons [39], and although this peak was more robust in W8UF than other treatment groups, it shifted toward a lower wave number (877 cm^−1^). This shows that W8UF had more C-H functional groups but suggests that a potential incomplete reaction led to an unstable structure.

**Figure 2:**
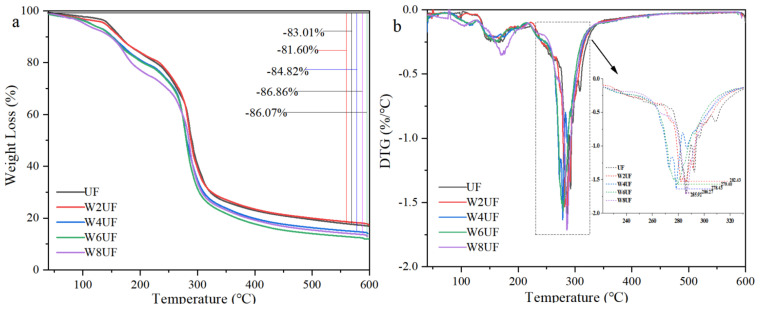
Thermo gravimetric (TG) (**a**) and derivative thermo gravimetric (DTG) (**b**) curves of the modified urea–formaldehyde adhesive, where UF, W2UF, W4UF, W6UF, and W8UF indicate treatments representing PVA additions in urea of 0%, 2%, 4%, 6%, and 8%, respectively.

TGA was performed to examine the influence of PVA modification on the thermal stability of PUF, and the thermal degradation (weight loss) of the samples traversed four stages with increasing temperature. The first stage (0–100 °C) evaporates free water, resulting in a mass loss of about 4%. The second stage (100–250 °C) causes the decomposition of low molecular weight substances such as bound water and methyl urea, resulting in a mass loss of about 18%. The third stage (250–350 °C) led to a mass loss of 60%, mainly due to the degradation of polymers in the resin and disintegration of the cross-linked structure [40] (Figure 2a). Using derivative thermogravimetric (DTG) analysis, it was evident that the maximum thermal degradation temperatures for UF, W2UF, W4UF, W6UF, and W8UF occurred at 286.3 °C, 282.4 °C, 278.4 °C, 279.4 °C, and 285.9 °C, respectively (Figure 2b). Compared to UF, the maximum temperatures of the PVA-treated samples were reduced, showing that thermal stability decreased for PUF. The final TGA stage (350–600 °C) caused the decomposition of methylene bonds and free formaldehyde release [37], and the residues of W4UF, W6UF, and W8UF were all lower than the UF control. Natural degradation has been shown to be the major cause of loss in resin materials [41], and lower residual PUF was beneficial to the natural degradation of the prepared mulch films. The mass loss of PUF was mainly due to the mass loss of PVA, which was significant compared to the thermal degradation of UF [42].

**Figure 3:**
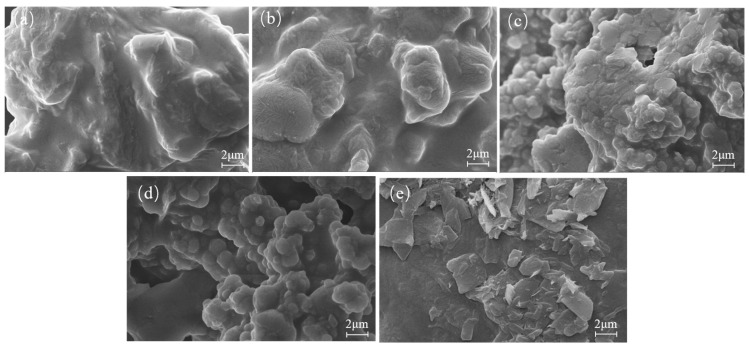
Scanning electron microscope (SEM) images of the modified urea–formaldehyde resin, where (**a**) UF, (**b**) W2UF, (**c**) W4UF, (**d**) W6UF, and (**e**) W8UF denote treatments representing PVA additions in urea of 0%, 2%, 4%, 6%, and 8%, respectively.

The structure and appearance of the films were evaluated using SEM (Figure 3a–e). Untreated UF resin exhibited an agglomerated structure without pores and uniform spherical particles (Figure 3a). An increasing number of protrusions was observed in the resin when 2% PVA was added (Figure 3b), and with increasing PVA, the particle size of the resin colloid decreased, forming a uniform spherical particle structure. The colloids with additions of 4% (Figure 3c) and 6% (Figure 3d) demonstrated this effect most significantly. However, when the addition reached 8%, transparent flake-like substances were observed to be precipitated on the surface of the resin (Figure 3e). Large agglomerations in the UF resins most likely resulted from colloidal aggregation [43], where the addition of PVA reduced the hydrogen bonding interactions between the polymer chains in the UF resin, and as the surface tension of the system decreased, the PUF colloidal particles rearranged, resulting in the changed structure [44]. The PUF particles formed a tightly packed structure due to hydrogen bonding, increasing the surface area and helping store and release nutrients, thus meeting growth needs [19]. UF and W2UF were difficult to degrade naturally due to their stable agglomerated structure, but the right amount of PVA (in this case, 4–6%) could form a PVA network with a low cross-linking density. Urea and formaldehyde interpenetrated into the PVA network during the synthesis of the urea–formaldehyde resin, where the compatibility of the polymers, as well as the uniform structural network, easily underwent thermal degradation [44]. This was consistent with the results obtained by TGA.

### 3.3. Mechanical Properties of PUF

**Figure 4:**
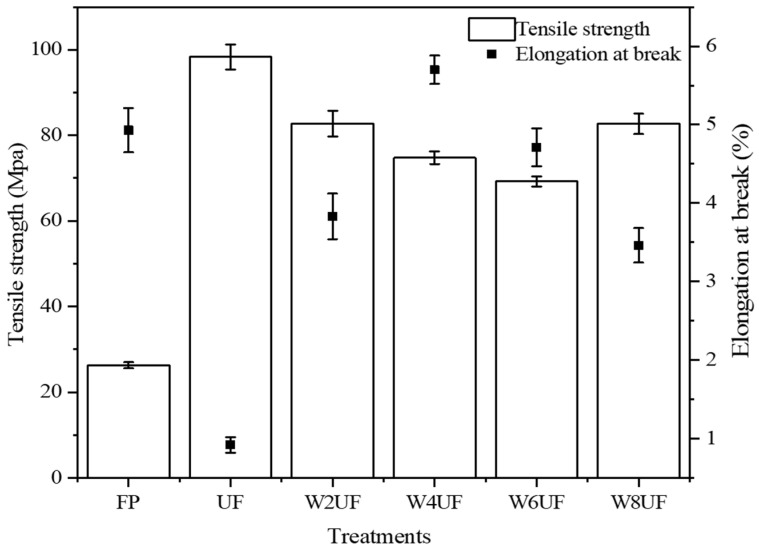
Tensile strength and elongation at break of the filter paper and modified urea–formaldehyde resin, where FP is the filter paper, and UF, W2UF, W4UF, W6UF, and W8UF denote treatments representing PVA additions in urea of 0%, 2%, 4%, 6%, and 8%, respectively.

After impregnation with UF, the tensile strength of the filter paper increased by 273.7%, and the elongation at break decreased by 435.9% (Figure 4) due to a more compact structure and the inherent brittleness of the UF resin cross-linked structural network [45]. When the PVA content increased, the tensile strength first decreased and then increased, while the elongation at break first increased and then decreased. In addition, the elongation at break reached a maximum when the PVA content was 4%. The improvement in tensile strength was attributed to the compacted FP structure resulting from the cured UF resin after impregnation, while the reduction in elongation at break of the filter paper was caused by the inherent brittleness due to the three-dimensional network structure of the UF resin. With the addition of PVA, the abundant hydroxyl groups in PVA reacted with formaldehyde to form polyvinyl formal, both of which are viscous substances. This led to a sharp increase in the viscosity of PUF, effectively preventing excessive penetration of the adhesive within the filter [46]. The decrease in viscosity and increase in tensile strength at 8% PVA is consistent with previous results (Table 1). The changes in elongation at break occurred because PVA is a water-soluble synthetic polymer rich in hydroxyl groups, and its pendant hydroxyl functional group has high activity. Thus, the addition of PVA as a plasticizer improved the intermolecular flexibility and chain mobility of the polymer chain, thereby reducing the brittleness of the resin and improving its durability [47]. However, the change in the mechanical properties of PUF is more related to its microscopic morphology. The addition of PVA enables UF to have a closed-cell flexible structure with high density and tiny cell diameter, a unique structure that increases resin durability [48]. In contrast, additional increases in PVA eventually caused the reaction with urea–formaldehyde to reach saturation, and excess PVA did not participate in the cross-linking structure reaction, causing the material to agglomerate (Figure 3e). As the degree of cross-linking decreased, the elongation at break decreased accordingly [45]. This is consistent with the structural changes seen in the SEM images. In summary, PUF with a PVA addition of 4% (W4UF) had the best performance, with a tensile strength of 74.8 MPa and an elongation at break of 5.7%.

### 3.4. Biodegradable Mulch Characteristics

**Figure 5:**
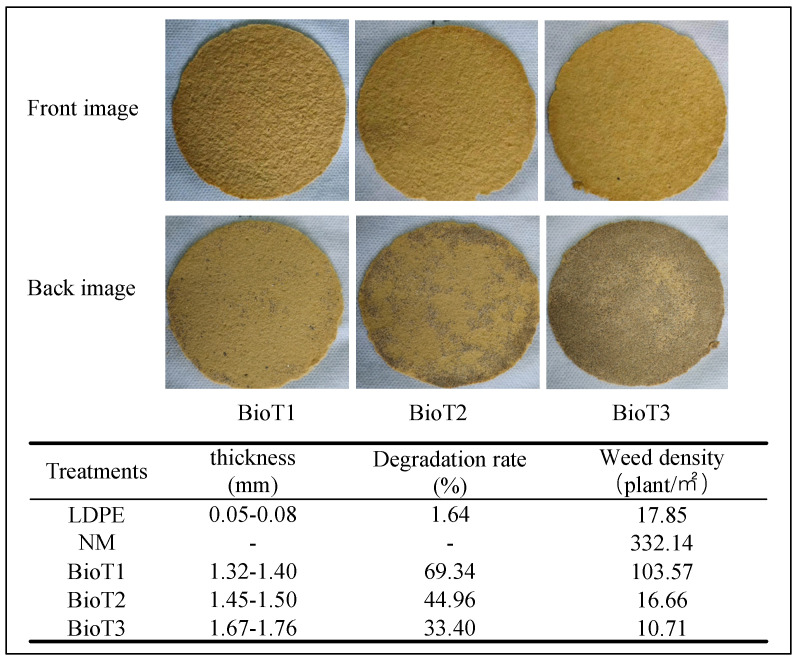
Photographs of the biodegradable films; thickness of the films; degradation rates of the films after 120 d buried in soil; and number of weeds per unit area. LDPE, NM, BioT1, BioT2, and BioT3 denote the low-density polyethylene, no mulching, and XP-water-PUF mass ratios of 1:30:2, 1:30:4, and 1:30:6, respectively.

The thickness of the biodegradable mulch films was 1.32–1.76 mm (Figure 5). With increasing PUF concentrations, the irregular protrusions on the surface of the biodegradable film gradually disappeared (front image), producing a more even surface and dense coating. This is because PUF was introduced as flexible particles into the fiber network of XP, which complemented each other to make the interior of the material more compact, thereby improving the density of the PUF-XP film. As can be seen in the back images, with increased PUF concentration, the amount of sand and soil bound by the film increased its thickness, meaning film thickness can be controlled by tuning the PUF addition, with higher thickness conducive to retaining soil moisture [49].

The biodegradability of the PUF-XP composite films was analyzed via soil burial testing. After being buried in the ground for 120 days, the degradation rate of LDPE film was only 1.64%, while the degradation rate of three PUF-XP films was 33.4–69.3% (Figure 5). Increasing PUF content decreased the degradation rate of films from 69.34% to 45.0% to 33.4%. Compared with the BBS and 2.0BBS/UF biodegradable films developed by the research group, the degradation rate of Bio T1 increased by 17.6% and 21.9%, respectively [33]. Degradation rate changes were associated with the film thickness, which increased with increasing PUF content, causing enhanced surface density of the film, making thicker films less susceptible to external factors incurred during the testing. Many grains of sand were observed adhering to the back of the membrane, and soil microorganisms for degradation were difficult to erode on short notice. Consequently, PUF-XP films could be chosen according to the cultivation cycles of different crops.

**Figure 6:**
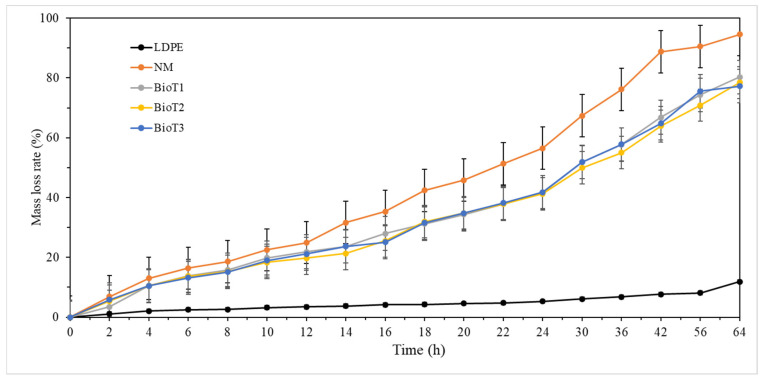
Mass-loss rates for soil moisture with mulch films, where LDPE, NM, BioT1, BioT2, and BioT3 denote the low-density polyethylene, no mulching, and XP-water-PUF mass ratios of 1:30:2, 1:30:4, and 1:30:6, respectively.

Figure 6 presents the water evaporation data for the various mulch treatments. After 64 h, the water evaporation rates for LDPE and NM were 11.9% and 94.6%, respectively. Compared to NM, the water evaporation rates for BioT1, BioT2, and BioT3 decreased by 14.3%, 16.2%, and 17.3%, respectively. Thus, the biodegradable composite film effectively inhibited water evaporation.

**Figure 7:**
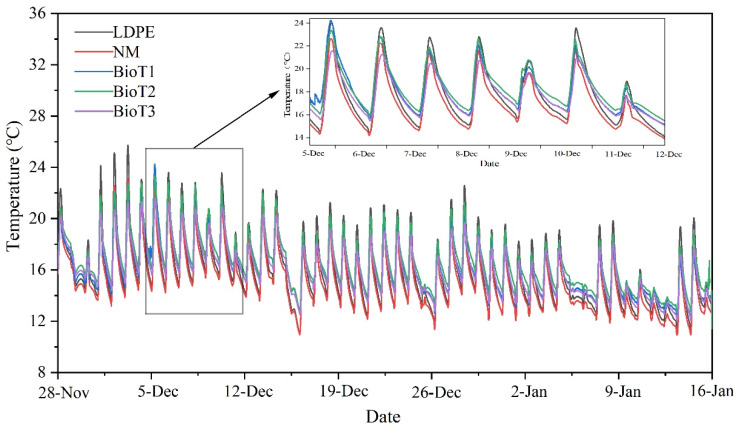
Temperatures at 10 cm soil depth under different mulching conditions. LDPE, NM, BioT1, BioT2, and BioT3 denote the low-density polyethylene, no mulching, and XP-water-PUF mass ratios of 1:30:2, 1:30:4, and 1:30:6, respectively.

After planting, weed density was calculated as the number of weeds per unit area (m^2^). Compared to no mulching (NM), the weed reduction rates of LDPE, BioT1, BioT2, and BioT3 were 94.6%, 68.8%, 95.0%, and 96.8%, respectively. Two of the biodegradable films showed excellent weed control, performing as good as or better than LDPE. This was due to the amount of PUF, which made BioT2 and BioT3 thicker and denser, allowing them to achieve successful weed growth inhibition [50]. BioT1 had the lowest weed control effect; however, it still inhibited more than 60% of the weed growth. Reduced weeds typically improve crop growth and yield due to reduced competition for light and nutrients. The ability of mulch to inhibit weeds is related to the amount of solar radiation entering the soil through the mulch, which was consistent with the changes in soil temperature observed during cultivation (Figure 7) [9]. Further, BioT2 and LDPE had the same temperature change rule. It should be noted that the symbiosis between some weeds and crops has been known to positively impact the ecological balance and activate beneficial microorganisms, which is beneficial to the growth of crops and root systems [51].

Changes in the soil temperature during the cultivation period were consistent between the different treatments (Figure 7). The temperature showed a general downward trend, consistent with an observed decrease in the external environment temperature. Upon inspection of the small temperature graph (Figure 7 inset), it can be seen that the maximum temperature for each treatment in order from high to low was LDPE, BioT2, BioT1, NM, and BioT3, while the order of minimum temperature followed BioT2, BioT1, BioT3, LDPE, and NM. LDPE recorded the highest temperatures during the day, as the film absorbed more solar radiation, and these higher temperatures could promote the early growth and flowering of crops. The biodegradable mulching films reduced the photosynthetically active radiation, resulting in cooler temperatures; however, they inhibited weed growth [52]. The highest temperature recorded for BioT3 was lower than that of NM, possibly because the dense membrane surface and greater thickness of BioT3 made it difficult for external heat to enter the soil. The temperature differences also reflected the heat storage capacity of the mulch. For example, the temperature difference between BioT2 and BioT1 varied less than LDPE and NM, indicating that BioT2 and BioT1 had a better insulation capacity than traditional LDPE mulch. Thus, higher soil temperatures and lower temperature fluctuations benefited plant growth and soil microbial activity [53].

### 3.5. Plant Growth Trial

Compared to other treatments, the cucumber root surface area density, root volume density, and root diameter significantly increased under LDPE mulching (Table 2). This was related to the higher water retention and weed inhibition properties of the LDPE film, as previous studies reported that these conditions were associated with increased root growth [54]. Among the three biodegradable mulch films, the specific root length and the root–shoot ratio increased with increasing PUF addition, while all other root indices decreased. The root length density, root surface area density, root volume density, root diameter, and specific root length of BioT1 were significantly higher than BioT2, BioT3, and NM and, compared to NM, increased 32.8%, 34.8%, 50%, 14.5%, and 13.4%, respectively. This was possibly due to the degradability and thermal insulation of the PUF mulching film, which may have positively affected the soil physicochemical properties and enhanced microbial activity to benefit cucumber root development [55].

**Figure 8:**
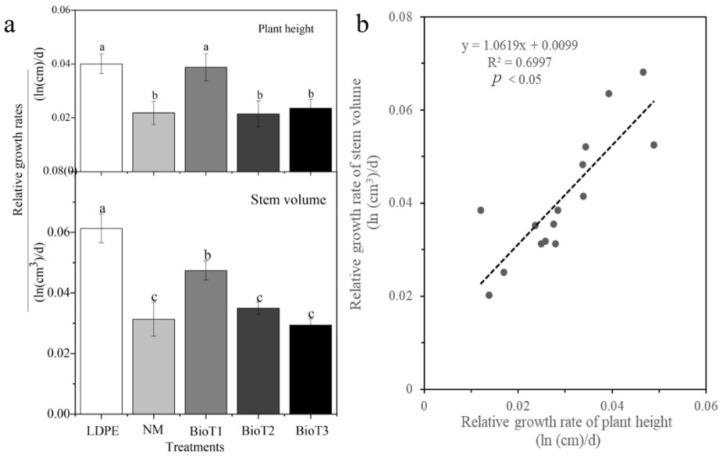
Relative growth rates (**a**) and linear relationship (**b**) of plant height and stem volume of cucumber plants covered with mulch films. The lowercase letters in the bars indicate significant differences among the treatments (*n* = 6, *p* < 0.05). LDPE, NM, BioT1, BioT2, and BioT3 indicate the low-density polyethylene, no mulching, and XP-water-PUF mass ratios of 1:30:2, 1:30:4, and 1:30:6, respectively.

As presented in Figure 8, the RGH-PH values of LDEP and BioT1 were higher than other treatments, with an increase of 45.3% and 43.5% compared to NM, respectively; however, there was no significant difference between BioT2, BioT3, and NM. Likewise, the RGH-SV of LDPE was higher than the other treatments, increasing by 49.0% over NM. With increasing PUF addition, the RGH-SV of the biodegradable films decreased, with BioT1 increasing by 34.1% compared to NM. Correlation analysis shows a significant, positive linear correlation between RGH-PH and RGH-SV, and LDPE had the highest RGH-PH and RGH-SV values. The main reason for this was that LDPE maintained high soil moisture and provided a suitable environment for crop growth [56]. BioT1 had a higher degradation rate than BioT2 and BioT3 (Figure 5), and it is likely that the nutrients released improved the soil properties, thereby stimulating plant growth [57].

**Figure 9:**
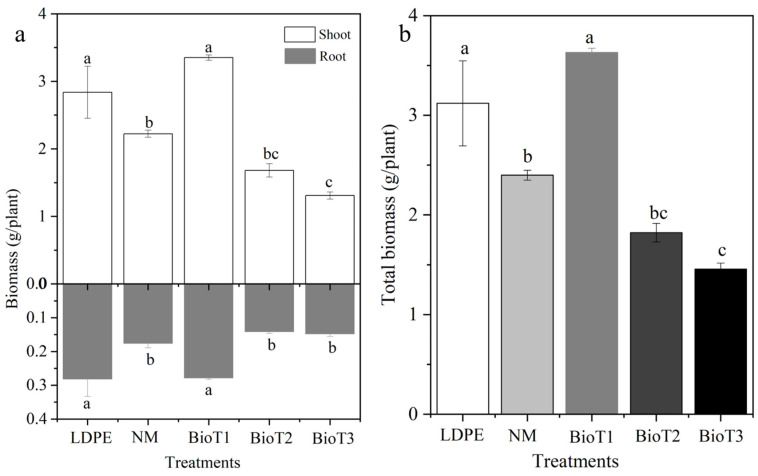
Aboveground (**a**), underground (**a**), and total biomass (**b**) of cucumber plants under different mulching conditions. The lowercase letters in the bars indicate significant differences among the treatments (*n* = 6, *p* < 0.05). LDPE, NM, BioT1, BioT2, and BioT3 indicate the low-density polyethylene, no mulching, and XP-water-PUF mass ratios of 1:30:2, 1:30:4, and 1:30:6, respectively.

Figure 9 presents both the above- and underground biomass, as well as the total biomass yields for each treatment. There was no statistical difference between LDPE and BioT1; however, they were significantly higher than the other treatments (Figure 9). It is interesting to note that aboveground and total biomass for BioT2 and BioT3 were less than NM. This is because the increased density of these two films attenuated root growth (Table 2). Also, the decrease in microbial activity decreased the availability of nutrients able to be absorbed by the cucumber [58]. It is also interesting that the total biomass of BioT1 was higher than LDPE and NM by 14.0% and 33.9%, respectively. The degradation of plant cellulose, lignin, and modified materials under the action of soil microorganisms results in the production of small organic molecules. Lignin derivatives (such as vanillin and ferulic acid) act as natural plant growth promoters, activating root peroxidase activity and enhancing the growth of cucumber underground parts. The degradation products of nanoscale cellulose fibers (20–50 nm in diameter) effectively improve soil aggregate structure, increase soil porosity, and significantly enhance water retention and aeration. Meanwhile, the slowly released organic carbon components from the paper film can chelate soil heavy metals, reducing the bioavailability of cadmium and lead, thereby alleviating plant heavy metal stress [59].

## 4. Conclusions

In this study, PVA was used as a modifier to enhance the strength and durability of UF and reduce the free formaldehyde content in the produced resin. The modified urea–formaldehyde resin (PUF) with a PVA content of 4% showed good compatibility and thermal degradation. The colloidal interpenetrating structural network formed by the PUF composites was beneficial for enhancing the mechanical properties of the films, and they offered storage and the release of nutrients. Using PUF as an auxiliary agent and XP as a filler, the biodegradable mulch films generated in this study created a cultivation environment similar to that provided by LDPE plastic film with good water retention and heat preservation performance. The degradation rate for BioT1 reached 69.34% after being buried in soil for 120 days, and during the cultivation of cucumber plants, the film inhibited 68.82% of weeds, thus promoting cucumber seedling growth and root development. Therefore, the biodegradable mulch film BioT1 could serve as an alternative to LDPE mulch film. However, how to industrialize biodegradable paper film is a new technological challenge that will be the focus of our future research.

## Figures and Tables

**Table 1:** Physical and chemical properties of the modified urea–formaldehyde resins.

Sample	pH Value	Viscosity /mPa·s	Solid Content /%	Curing Time/s	Free Formaldehyde Content/%	Water Absorption/%
W6UF	8.17 ± 0.01 ^a^	78.47 ± 0.17 ^a^	41.16 ± 0.08 ^d^	70.0 ± 0.9 ^b^	0.290 ± 0.001 ^c^	38.3 ± 1.7 ^a^
W4UF	7.97 ± 0.01 ^b^	67.93 ± 0.03 ^b^	43.95 ± 0.19 ^c^	67.0± 0.7 ^c^	0.260 ± 0.003 ^d^	39.8 ± 2.7 ^a^
W2UF	8.02 ± 0.04 ^ab^	23.67 ± 0.09 ^d^	47.09 ± 0.16 ^b^	63.0 ± 0.4 ^d^	0.310 ± 0.005 ^b^	40.6 ± 1.3 ^a^
W8UF	8.09 ± 0.05 ^ab^	45.80 ± 0.15 ^c^	39.01 ± 0.11 ^e^	74.0 ± 0.6 ^a^	0.290 ± 0.004 ^c^	41.5 ± 0.6 ^a^
UF	7.77 ± 0.04 ^c^	18.57 ± 0.03 ^e^	49.76 ± 0.06 ^a^	56.0 ± 0.3 ^e^	0.370 ± 0.003 ^a^	24.2 ± 0.6 ^b^

Note: Different lowercase letters in the same column indicate significant differences among the treatments (*n* = 3, *p* < 0.05), where UF, W2UF, W4UF, W6UF, and W8UF indicate treatments representing PVA additions in urea of 0%, 2%, 4%, 6%, and 8%, respectively.

**Table 2:** Influence of different treatments on the morphological characteristics of cucumber plant roots.

Treatment	Root Length Density/(cm·cm^−3^)	Root Surface Area Density/(cm^2^·cm^−3^)	Root Volume Density /(cm^3^·cm^−3^)	Root Diameter /(mm)	Specific Root Length/(m·g^−1^)	Root Shoot Ratio
LDPE	0.382 ± 0.005 b	0.101 ± 0.009 a	0.0090 ± 0.0006 a	0.75 ± 0.02 a	47.2 ± 2.0 c	0.188 ± 0.021 bc
NM	0.283 ± 0.010 c	0.058 ± 0.003 b	0.0020 ± 0.0003 c	0.63 ± 0.03 b	57.8 ± 3.7 ab	0.164 ± 0.002 c
BioT1	0.421 ± 0.010 a	0.089 ± 0.005 a	0.0040 ± 0.0004 b	0.74 ± 0.02 a	66.7 ± 3.1 a	0.214 ± 0.021 abc
BioT2	0.24 ± 0.008 d	0.047 ± 0.002 b	0.0010 ± 0.0001 cd	0.60 ± 0.03 b	51.6 ± 3.7 bc	0.246 ± 0.006 a
BioT3	0.140 ± 0.007 e	0.028 ± 0.001 c	0.0010 ± 0.0001 d	0.67 ± 0.03 ab	33.5 ± 1.5 d	0.231 ± 0.018 ab

Note: Different lowercase letters in the same column indicate significant differences among the treatments (*n* = 6, *p* < 0.05). LDPE, NM, BioT1, BioT2, and BioT3 denote the low-density polyethylene, no mulching, and XP-water-PUF mass ratios of 1:30:2, 1:30:4, and 1:30:6, respectively.

## Data Availability

No data were used for the research described in the article.
